# A Chaotic Compressive Sensing Based Data Transmission Method for Sensors within BBNs

**DOI:** 10.3390/s22155909

**Published:** 2022-08-07

**Authors:** Wei Wu, Haipeng Peng, Fenghua Tong, Lixiang Li, Binzhu Xie

**Affiliations:** 1 Information Security Center, State Key Laboratory of Networking and Switching Technology, Beijing University of Posts and Telecommunications, Beijing 100876, China; 2 Shandong Provincial Key Laboratory of Computer Networks, Shandong Computer Science Center (National Supercomputer Center in Jinan), Qilu University of Technology (Shandong Academy of Sciences), Jinan 250014, China

**Keywords:** compressive sensing, information concealment, chaos theory

## Abstract

Body to body networks (BBNs) are a kind of large-scaled sensor network that are composed of several wireless body area networks (WBANs) in the distributed structure, and in recent decades, BBNs have played a key role in medical, aerospace, and military applications. Compared with the traditional WBANs, BBNs have larger scales and longer transmission distances. The sensors within BBNs not only transmit the data they collect, but also forward the data sent by other nodes as relay nodes. Therefore, BBNs have high requirements in energy efficiency, data security, and privacy protection. In this paper, we propose a secure and efficient data transmission method for sensor nodes within BBNs that is based on the perception of chaotic compressive sensing. This method can simultaneously accomplish data compression, encryption, and critical information concealment during the data sampling process and provide various levels of reconstruction qualities according to the authorization level of receivers. Simulation and experimental results demonstrate that the proposed method could realize data compression, encryption, and critical information concealment for images that are transmitted within BBNs. Specifically, the proposed method could enhance the security level of data transmission by breaking the statistical patterns of original data, providing large key space and sensitivity of the initial values, etc.

## 1. Introduction

With the thriving of technologies that relate to telecommunications, sensors and networks, the traditional internet with servers, personal computers, and cell phones acting as interconnection entities have been gradually transforming into the Internet of things (IoT), which connects vehicles, electric appliances, even human bodies and aims to realize the interconnection of everything. In order to achieve telemedicine, wireless body area networks (WBANs), for example, which represent the internet of human beings, implant specific sensors on the surface of or even in the body of individuals [1,2,3,4].

Recently, apart from medical service and health care, WBANs also have the possibility of applications in military and aerospace fields, such as being applied to monitor vital signs of soldiers or astronauts and to implement first aid treatments. During routine training, WBANs could guard trainees by means of collecting training data and adjusting the training intensity. In military actions, WBANs could assist in developing attackers and defenders’ strategies through monitoring body postures of both our armies and the opponents. On battlefields, WBANs could contribute to communications between soldiers as well as to data deliveries to commanders in the base. In addition, WBANs could provide help in monitoring physical status and positions of soldiers by embedding monitor sensors into military uniforms. Generally speaking, WBANs play an essential role in enhancing the accuracy, survivability, and connectivity in virtually every aspect of military operations. In addition, WBANs could be deployed along with astronauts, and by equipping space capsules with WBAN sensors, important data that concern space research as well as physical status information are transmitted from space to control centers through satellite channels. Such space applications broaden the scope where WBANs may exert themselves.

The WBAN sensors embedded in human bodies can gather a wide range of vital signs, such as heart rates, blood pressures, electroencephalograms (EEGs), electrocardiograms (ECGs), and so forth [5], and some compressive sensing based methods have already been proposed to deal with such signals [6,7]. The WBAN sensors send data they collect to monitoring centers, and thus distant diagnosis could be carried out and proper solutions may be proposed based on the data received. Because the transmission distances of WBAN sensors are limited, commonly, WBANs need the aid of additional typical network infrastructures to transmit data. For example, sensor nodes within WBANs that are distributed in various parts of individual bodies first send the data they collect to mobile phones, computers, satellites, or other types of terminals, and then these terminals transmit these data to monitoring centers or remote databases through Internet or satellite channels. WBANs are also sought after as they have expanded the service scope of traditional medical services that need specific time and workplaces and helped to realize a real-time and mobile mode of medical services.

Apart from limited transmission distances, the WBANs that concern military or space technologies and medical applications often have a limited number of participant entities. When accompanied with the extension of application scopes, being applied in occasions such as major sport events, military actions, or disaster rescues, for instance, WBANs are required to be transferred from centralized data communication modes to distributed ones, and therefore body to body networks (BBNs) are gradually formed. BBNs can be regarded as extended WBANs, that is, multiple WBANs are interconnected with each other to generate a BBN. As a result, entities within WBANs could not only interact with other nodes in the same WBAN, but could also communicate across different WBANs, namely, WBANs can communicate with each other.

**Figure 1:**
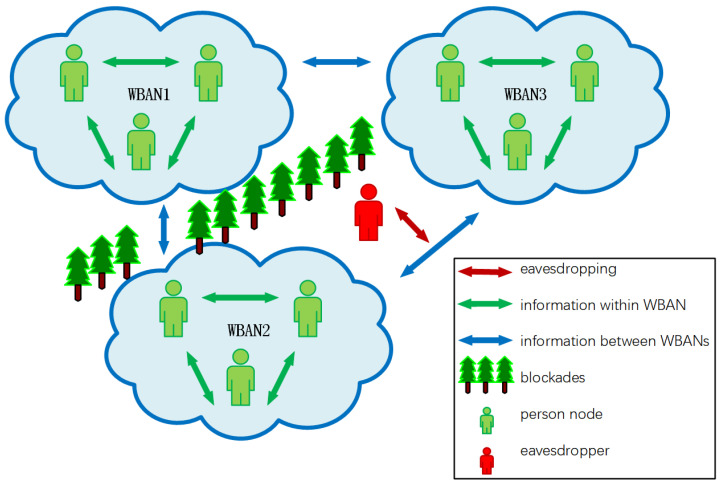
The network structure of a BBN. Each WBAN consists of a number of person nodes with sensors on or in their bodies. Three WBANs form a BBN and could interact with one another.

As shown in Figure 1, a BBN that consists of three WBANs is used to monitor vital signs of human beings, battlefield environment data, enemy intelligence, and other information, and such information could be exchanged between WBANs or to be sent to remote control centers. At the same time, in the light of the control signals and feedback commands that are transmitted from remote control centers, soldier nodes could be timely administered and arranged, so that efficient military operations are realized. The data gathered by BBNs vary along with the changes in environment and location. The information monitored by BBN sensors, such as human postures and vital signs, is sent by wireless networks and contributes to drawing up operation plans and making decisions, for example, timely evacuation commands could be instructed beforehand as a result of certain discoveries found in data transmitted by BBNs, so as to avoid unnecessary casualties or economic loss.

Moreover, compared with WBANs, BBNs often have larger network scales, longer transmission distances, and wider transmission ranges. Sensor nodes within BBNs not only transmit data caught by themselves, but also forward data collected by other nodes, and such a process causes massive energy consumption. However, BBN sensors are frequently battery-powered, and batteries that are installed on the surface of human bodies or even embedded in the body of individuals are inconvenient to be recharged or replaced. When battery power is exhausted, BBN nodes tend to lose efficiency. Therefore, it is critical to settle energy saving issues when designing data transmission schemes within BBNs, and a practical measure could be to reduce the amount of communication data by compressing the data to be transmitted.

Another difficulty to be overcome is to address eavesdropping problems within BBNs. Because BBNs commonly use wireless transmission technology, network links throughout BBNs may be open and vulnerable to eavesdropping. In particular, such networks are often teeming with private data related to human bodies or confidential information concerning militaries, space technologies, etc. Therefore, in the process of BBN data transmission, an encryption scheme is a must to prevent eavesdroppers from obtaining sensitive information so that the security level of data transmission is enhanced. Furthermore, in order to avoid the potential risk brought by the ownership of a master key that is solely possessed by a single node in the network of the cryptosystem, a certain security protection mechanism is needed. For example, it is necessary to ensure that the secret information carried by original signals will not be disclosed when the person who carries critical data is arrested by enemies. A feasible method is concealing critical information in original signals by means of hierarchical authorization for multilevel receivers. At present, the encryption and compression processes of common schemes are independent. Another issue is, when processing images or videos by sensor nodes within BBNs or WBANs, their storage or computing resources barely meet the requirements of the energy, computing, or other resource consumption level brought by the introduction of classical encryption algorithms. How to accomplish data compression, encryption, and critical information concealment efficiently becomes a burning issue to be addressed.

## 2. Related Work

This section briefly summarizes the medical and military applications of BBNs, resource consumption and security problems faced by sensor nodes in BBNs, and recent progress in the research of compressive sensing (CS) theory.

In recent years, BBNs have already developed a number of applications for medical services and health care. The CodeBlue project launched by Harvard University attempted to achieve multi-hop transmission through routing nodes in WBANs [8]. A project called the Advanced Health and Disaster Aid Network [9], which applied WBANs in disaster rescues, could only allow a rather limited number of sensors taking part in the communication process due to restrictions of the bandwidth. A. Milenkovic et al. proposed a Wearable Health Monitor System [10], in which a large-scale WBAN for health monitoring was deployed, but the performance of this system was impacted by energy consumption issues.

There are also a number of research achievements in military applications of BBNs. At Walter Reed Army Medical Center (WRAMC), research on supplementary treatments involving cell phones as tools for diabetes treatment was conducted [11], and this could have a profound impact on the outcome of remedies for the elderly and patients with diabetes or other chronic diseases by introducing WBANs in remote health monitoring. Emeka E. Egbogah et al. proposed a cost-efficient data transmission method to meet the demands of monitoring soldiers’ vital signs [12]. This method reduced the energy consumption of WBANs worn by soldiers through the means of formulating and solving two optimization problems.

Currently, WBANs and BBNs offer new possibilities for improving the performance of individuals and teams in terms of military operations. For instance, WBANs could play a fundamental role in preventing critical information from being stolen by enemies [13]. In this paper, in order to avoid threats generated by the single node problem, a group of sensors were deployed to collect important information about the circumstances and nearby new actions, and, at the team level, the information gathered by sensors could enable commanders to coordinate tasks with team members. Singh D. et al. visualized a military health service platform and designed a model based on semantic edge [14]. Salayma M et al. proposed a new military medical application that could assess the level of soldier fatigue and combat readiness, so as to protect staff in uniform [15].

Aiming at secure and efficient data transmission within BBNs, some solutions are raised. Several energy harvesting methods are proposed in [16,17,18,19]. Energy harvesting means that nodes within BBNs collect or generate power from human bodies or other sources to supplement the batteries of sensors. The batteries may be charged by bioenergy or energies generated from body heat, vibration, or friction of movement, etc. However, such energy harvesting functions always add specific circuits to the hardware of BBN sensors, such as energy collectors or power management circuits. In this way, the costs of sensor nodes are increased, which may be detrimental to widespread deployment of BBNs. For energy saving, Zhang C et al. proposed a novel medium access control (MAC) protocol with the function of reducing power consumption [20]. In [21,22,23], efficient routing protocols suitable for BBNs were designed, and several energy optimization and control algorithms were proposed in [24,25].

To account for security, there have been several proposed schemes. S. Al-Janabi et al. presented a solution for encryption and authentication processes in the link layer of BBNs and proposed a security suite based on IEEE 802.15.6 standard [26]. X. Liu et al. offered an information security management system for WBANs to ensure data confidentiality and integrity [27]. A key generation method that introduced attributes of wireless channels of BBNs was raised in [28]. L. Wu et al. purveyed an anonymous authentication method for BBNs, which could resist man-in-the-middle attacks [29]. Finally, in [30], H. Zhu et al. applied homomorphic encryption to BBNs to realize data collection and query without the neglect of privacy protection.

The following paragraphs contain a brief introduction of the main research progress in (compressive sensing) CS. CS theory is a signal sampling and compression theory that was first proposed by Tao in 2006 [31,32,33]. Once put forward, this theory has been widely used in telecommunications, networks, signal processing, radars, aviation, biomedical applications, etc. CS does not merely address the problem of data compression. It also achieves data encryption simultaneously. Such characteristics may meet the requirements of data compression and encryption of sensor networks. Noticeably, CS can realize data compression and encryption in solely one step [34,35,36]. Peng et al. improved the generation process of the measurement matrix and enhanced the security level of data transmission by introducing chaotic systems, but concealing critical information was not considered [37]. Mehmet Yamaç et al. combined CS and data hiding, although their scheme has not achieved the acme of perfection in terms of resisting statistical attacks [38].

To the best of our knowledge, research on BBNs commonly considers data security, energy efficiency, and critical information concealment issues separately. Although a proportion of existing CS based schemes could accomplish data encryption and compression at the same time, or could realize efficient data transmission to a certain extent, they did not address the problems that concerns critical information concealment.

According to the characteristics of BBNs and the above-mentioned issues to be settled, this paper proposes a secure and efficient data transmission method based on the chaotic CS model and there are three main contributions.

(1) For the purpose of achieving energy efficiency, it should be considered that the capacity of batteries installed on BBN sensors is fairly limited, and recharging or replacing these batteries are often not convenient, especially when the batteries have been implanted into human bodies. And because of the expansion of network scales, the complexity of natural or external environments, or the huge volume of data to be transmitted, power of BBN batteries may consume fast. So, energy saving issues become a must while designing data transmission schemes applied in BBNs. Based on CS theory, this paper gives an efficient data transmission method, which completes data compression, encryption and critical information concealment simultaneously.

(2) For the purpose of realizing transmission security, it should be considered that the majority of data transmitted in BBNs may contain confidential sections, especially when the data concern vital signs of human beings or personal information. Especially, while being applied to medical care or military affairs, BBNs highly probably transmit a substantial amount of data involving critical or private information. On the one hand, under complex circumstances of the real world, open links may be vulnerable to be eavesdropped, which may lead to critical information leaking or other problems. Moreover, even if data are transmitted solely in internal channels, secure data transmission scheme is also imperative, since data may be forwarded several times and these processes may generate many copies of original signals involving critical information. Based on chaos theory, this paper designs a novel secure data transmission method, which could enhance the security level of data transmission by breaking the statistical patterns of original data, providing large key space and sensitivity of the initial values, etc.

(3) For the purpose of enhancing flexibility, the proposed method considers from two aspects. For data senders, the proposed method can flexibly control the proportion and the quantity of sections to be concealed which may contain critical information, according to different scenarios and application requirements. For example, the transmitted information can be concealed completely by senders without affecting the data recovery quality. In addition, senders could select independently only one or more sections to be concealed. For the data receivers, the information they could obtained varied according to their authorization levels. For instance, the receivers with the restricted authorization could only achieve the very part of data without critical information, while the receivers with the full authorization could recovery nearly intact original information.

## 3. Preliminaries

### 3.1. Compressive Sensing

Compressive sensing is a signal processing method that was proposed in [31]. It represents original signals in dimensionally reduced values that are called observational values. Suppose the original signal is $s\in R^{N}$, and it has a sparse compressible representation in a basis $\Psi \in R^{N\times N}$, that is x=Ψs, where $x\in R^{N}$ is a *k*-sparse vector, namely, there is at most *k* of its entries that are nonzero, and in this paper we consider that Ψ is an orthogonal matrix ($\Psi \Psi^{T}=I_{N\times N}$,$\Psi^{T}\Psi =I_{N\times N}$). The compressive sensing process is then taken as

(1) y=As

where $A\in R^{M\times N}$ (M<N) is the measurement matrix and $y\in R^{M}$ consists of measurement values, also named observational values.

We then obtain

(2) $$y=A\Psi \Psi^{T}s=A\Psi^{T}x=\Phi x$$

where the matrix $\Phi =A\Psi^{T}$ is the sensing matrix, and the sensing matrix should satisfy the condition proposed by Candès and Tao in [32,33].

A matrix Φ satisfies the restricted isometry property (RIP) of order *k* if there exists a $\delta_{k}\in \left(0,1\right)$ such that

(3) $$\left(1-\delta_{k}\right){∥x∥}_{2}^{2}\leq {∥\Phi x∥}_{2}^{2}\leq \left(1+\delta_{k}\right){∥x∥}_{2}^{2}$$

holds for all $x\in \sum_{k}$, where $\sum_{k}=\left\{x:{∥x∥}_{0}\leq k\right\}$ denotes the set of all *k*-sparse vectors in $R^{n}$.

From Equation (3), we can also infer that, satisfying the rule of RIP with order 2k, the measurement matrix Φ approximately preserves the distance between any two *k*-sparse vectors, which is essential to noise robustness [39].

There are many methods to reconstruct the original signal *s* from the measurement values, and orthogonal matching pursuit (OMP) [40] is one of the simplest greedy approaches to accomplish CS reconstruction. The OMP algorithm first finds the column of *A* that is most correlated with the measurements and then repeats this step by correlating the columns with the residual signal, which is achieved by subtracting the contribution of a partial estimate of the signal from the original measurement vector.

### 3.2. Chaotic System and Chaotic Compressive Sensing

Chaos, which is also called non-linear dynamics, is a seemingly irregular movement with internal randomness that occurs in a deterministic system. Chaotic systems are characteristic of internal randomness, sensitive dependence on initial conditions, boundedness, aperiodicity, and ergodicity. Tent and logistic systems are two typical chaotic systems and are defined as follows.

Based on tent system Equation (4), we can get a chaotic sequence $z_{l}^{{}^{\prime }},l=1,2,3\cdots$,

(4) $$z_{l+1}^{{}^{\prime }}=\left\{\begin{array}{c} z_{l}^{{}^{\prime }}/b,\;\;0<z_{l}^{{}^{\prime }}<b\\ \left(1-z_{l}^{{}^{\prime }}\right)/\left(1-b\right),b<z_{l}^{{}^{\prime }}<1 \end{array}\right.$$

where b,0<b<1 is the chaotic parameter and $z_{0}^{{}^{\prime }}$ is an initial value.

Simultaneously, another chaotic sequence $z_{l},l=1,2,3\cdots$, with the chaotic parameter μ and an initial value $z_{0}$ as inputs, could be generated by logistic system Equation (5),

(5) $$z_{l+1}=\mu z_{l}\left(1-z_{l}\right),\mu \in \left(0,4\right]$$

As we noted above, the compressive sensing measurement matrices should be chosen specifically, in order to guarantee the quality of reconstruction. L. Yu et al. presented that chaotic matrices could satisfy RIP and perform as well as Gaussian random matrices and Bernoulli random matrices when they act as compressive sensing measurement matrices [41].

## 4. Proposed Method

**Figure 2:**
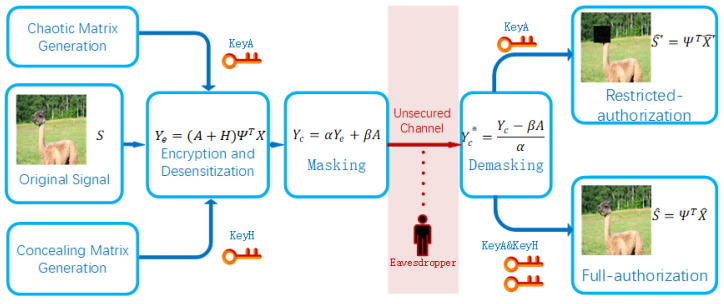
The main workflow of the proposed method. The left side shows the data compression, encryption, and concealing and masking processes done by a sender. The right side shows the data demasking, decryption, and retrieving processes done by receivers. The red part in the middle represents an open or insecure channel that might leak information to eavesdroppers.

This section presents the details of the proposed method, and Figure 2 illustrates its main processing procedures. On the senders’ side, to begin with, a chaotic matrix is generated, which acts as the CS measurement matrix. At the same time, a concealing matrix is generated according to the critical sections of the original signal. Next, CS is processed in order to accomplish data compression, encryption, and critical information concealment through a single step. Last, the encrypted and concealed signal is masked by a chaotic matrix for the preparation of transmission through an open or insecure channel. On the receivers’ side, reconstruction procedures are processed by restricted-authorized receivers and full-authorized receivers separately. Restricted-authorized receivers who merely possess key A can obtain signals with critical sections concealed. Full-authorized receivers who possess both key A and key H can achieve the entire original signals that contain critical information. In addition, it should be noted that eavesdroppers could obtain little useful information, even if they manage to catch the processed signals by some malicious means.

As shown in Figure 2, there are four roles in the proposed method.

- Senders process data compression, encryption, and concealment procedures based on CS theory and then mask the processed data to be transmitted. A typical sender in the proposed method could be a sensor within BBNs, which is possibly mobile electronic equipment linked to local area networks (LANs) or wide area networks (WANs).
- Receivers with restricted authorization only possess key A, that is to say, they could merely reconstruct the portions of the original signals without critical information.
- Receivers with full authorization possess both key A and key H, in other words, they could realize the original signals that contain critical information after the reconstruction procedure.
  Characteristically, receivers in the proposed method could be sinks or fusion nodes within BBNs or servers that may be deployed in data centers, etc.
- Eavesdroppers may listen to the transmission channels for the purpose of catching available information. Attackers between senders and receivers, for example, are likely to intercept network flows and obtain all or just part of transmitted data.

The details of operations are as follows.

### 4.1. On the Senders’ Side

The operations of senders enable data compression, encryption, critical information concealment, and masking before data transmission.

#### 4.1.1. Generation of Chaotic Matrices

Chaotic sequences are used to generate measurement matrices and concealing matrices, and, to increase the security level of the proposed method, we use two heterogeneous chaotic systems to generate measurement matrices and concealment matrices separately. Here, we choose the tent system for measurement matrix generation and the logistic system for concealing matrix generation. Other chaotic systems, the Chebyshev system for instance, can also be used to generate such deterministic matrices.

Based on tent equation Equation (4), we get a chaotic sequence $z_{l}^{{}^{\prime }},l=1,2,3\cdots$. Simultaneously, another chaotic sequence $z_{l},l=1,2,3\cdots$ could be generated by logistic equation Equation (5).

Next, a measurement matrix teeming with chaotic sequences could be generated with the following two steps:

Step 1: After the chaotic sequence $z_{l}^{{}^{\prime }}$ is generated, we sample this sequence using the sampling initial position $n_{0}$ and sampling distance *d*. Therefore, the sampled sequence $x_{n}^{{}^{\prime }}$ is obtained as Equation (6).

(6) $$x_{n}^{{}^{\prime }}=z_{n_{0}+nd}^{{}^{\prime }},n=1,2,3\cdots$$

Step 2: We arrange the elements in the sampled sequence to generate a matrix whose order is M×N with some mapping relationships, and the formed matrix can be used as the CS measurement matrix. The following mapping function is available, but is not limited to,

(7) $$a_{ij}=x_{i(n-1)+j}^{{}^{\prime }},n=1,2,3\cdots$$

where the measurement matrix is $A\in R^{M\times N}$ (M<N) and $a_{ij}$ is the entity in matrix *A* with a position of row ith and column jth.

Similarly, a concealing matrix could be generated based on chaotic sequence $z_{l},l=1,2,3\cdots$. Suppose $s\in R^{N}$ is a k-sparse discrete signal that contains critical information, and *j* indicates the row number of elements in *s*. We define a set *C* that consists of the critical entities that are contained in *s* and then can get a concealing matrix *H* using the function as below:

(8) $$h_{ij}=\left\{\begin{array}{c} 0,\;\;if\;s_{j}\notin C\\ -z_{i}*a_{ij},if\;s_{j}\in C \end{array}\right.$$

where the concealing matrix is $H\in R^{M\times N}$ and $h_{ij}$ is the entity in *H* with a position of the *i*th row and the *j*th column.

#### 4.1.2. Data Compression, Encryption, and Critical Information Concealment

The step given in Equation (9) simultaneously completes the processes including data compression, encryption, and critical information concealment.

(9) $$y_{e}=\left(A+H\right)s$$

where $y_{e}\in R^{M\times 1}$ is the compressed, encrypted, and concealed signal.

Easily, it can be deduced that,

(10) $$y_{e}=\left(A+H\right)\Psi^{T}x=\Phi^{*}x$$

where $\Phi^{*}=\left(A+H\right)\Psi^{T}$ acts as the sensing matrix that will be used to reconstruct the signal *x* through the OMP algorithm by receivers with full authorization.

We can also rewrite Equation (9) as below:

(11) $$y_{e}=A\Psi^{T}x+H\Psi^{T}x=\Phi x+\epsilon$$

where $\epsilon =H\Psi^{T}x$ denotes noise. The matrix $\Phi =A\Psi^{T}$ acts as the sensing matrix that will be used to reconstruct the signal *x* through the OMP algorithm by receivers with restricted authorization.

#### 4.1.3. Masking

A chaotic masking method is introduced to ensure the processed signal meet mainstream security benchmarks concerning image encryption. As a result, the proposed method could prevent malicious attacks to some extent by breaking the statistical patterns of original data, providing large key space and sensitivity of the initial values, etc.

After the compressed, encrypted, and concealed signal $y_{e}$ is obtained, we mask it with a chaotic sequence before data transmission. Here the matrix *A*, which is generated by Equation (7), is suggested to be partially reused for masking with the consideration to save computing power and energy consumption.

The masking process is shown in Equation (12).

(12) $$y_{c}=\alpha y_{e}+\beta a$$

where parameters α, β are added to adjust masking magnitud, *e* and *a* is a column of matrix *A*. Apparently, the chaotic matrix *A* can be expanded using the chaotic sequence according to the method depicted in Equation (6) if the columns are exhausted when masking.

### 4.2. On the Receivers’ Side

The operations done by receivers rely on secret keys that contain generation information of matrix *A* and matrix *H*. The reconstruction processes of original signals with and without critical information are carried out, respectively, by full-authorized receivers and restricted-authorized receivers. Restricted-authorized receivers could merely obtain the data without critical sections, whereas the full-authorized receivers could recover the complete data.

#### 4.2.1. Receivers with Restricted Authorization

For restricted-authorized receivers, they only possess key A that contains parameters to generate the measurement matrix *A*, and this key behaves as a symmetric decryption key. The details of the data reconstruction process are shown as below.

Step 1: To begin with, from Equations (11) and (12), we can get the transformed encrypted and concealed signal $y_{c}^{*}\in R^{M\times 1}$ as Equation (13).

(13) $$y_{c}^{*}=\frac{y_{c}-\beta a}{\alpha }=\Phi x+\epsilon .$$

Step 2: The OMP algorithm could be exploited to obtain the estimate of $x^{\prime }$, and here $x^{\prime }$ represents the concealed form of *x*.

(14) $$\overset{\wedge }{x^{\prime }}=arg\underset{x}{min}{∥x∥}_{1}\;subject\;to\;y_{c}^{*}=\Phi x=A\Psi^{T}x$$

Step 3: Finally, an inverse transformation of x=Ψs is performed to obtain the estimate of the concealed form of $s^{\prime }$.

(15) $$\overset{\wedge }{s^{\prime }}=\Psi^{T}\overset{\wedge }{x^{\prime }}$$

#### 4.2.2. Receivers with Full Authorization

For fully authorized receivers, they not only possess key A that contains parameters to generate the measurement matrix *A*, but also possess key H that contains parameters to generate the concealing matrix *H*, and these two keys work together as symmetric decryption keys. The details of data reconstruction process are shown as below.

Step 1: To begin with, from Equations (10) and (12), we can get the transformed encrypted and concealed signal $y_{c}^{*}\in R^{M\times 1}$

(16) $$y_{c}^{*}=\frac{y_{c}-\beta a}{\alpha }=\Phi^{*}x.$$

Step 2: The OMP algorithm could be exploited to obtain the estimate of *x*.

(17) $$\overset{\wedge }{x}=arg\underset{x}{min}{∥x∥}_{1}\;subject\;to\;y_{c}^{*}=\Phi^{*}x=\left(A+H\right)\Psi^{T}x$$

Step 3: Finally, an inverse transformation of x=Ψs is performed to obtain the estimate of the original signal *s*.

(18) $$\overset{\wedge }{s}=\Psi^{T}\overset{\wedge }{x}$$

Eavesdroppers may manage to complete the same operations as normal receivers after they capture transmission data that are processed by the proposed method. However, because they do not possess secret keys, which act as essential resource for reconstruction, they cannot obtain the available information sent by senders. When considering the key distribution issue that could be applied in IoT, there are several existing solutions [42,43,44]. Therefore, such issues are not discussed in this paper.

## 5. Feasibility Analysis

This section contains two parts. First, we demonstrate the feasibility of the proposed method theoretically. Because the reconstruction process of full-authorized receivers is rather similar to the reconstruction process of traditional compressive sensing, in this section, we only discuss the feasibility of the reconstruction of restricted-authorized receivers. In the second part, we conduct experiments to verify that the proposed method is practically feasible.

To explicitly explain the processes of critical information concealment and retrieval, we expand Equation (9) in the following way, and assume that $s_{p}$ is the element that involves critical information.

$\left[\begin{array}{c} y_{e1}\\ y_{e2}\\ y_{e3}\\ \vdots \\ y_{eM} \end{array}\right]=\left(\left[\begin{array}{ccccc} a_{11} & a_{12} & a_{13} & \cdots & a_{1N}\\ a_{21} & a_{22} & a_{23} & \cdots & a_{2N}\\ \vdots & \vdots & \vdots & \ddots & \vdots \\ a_{M1} & a_{M2} & a_{M3} & \cdots & a_{MN} \end{array}\right]+\right.$

(19) $$\begin{array}{c} \left.\left[\begin{array}{cccccc} 0 & 0 & \cdots & -z_{1}*a_{1p} & \cdots & 0\\ 0 & 0 & \cdots & -z_{2}*a_{2p} & \cdots & 0\\ \vdots & \vdots & \vdots & \vdots & \ddots & \vdots \\ 0 & 0 & \cdots & -z_{M}*a_{Mp} & \cdots & 0 \end{array}\right]\right)\left[\begin{array}{c} s_{1}\\ s_{2}\\ \vdots \\ s_{p}\\ \vdots \\ s_{N} \end{array}\right]\\ =\left[\begin{array}{cccccc} a_{11} & a_{12} & \cdots & \left(1-z_{1}\right)*a_{1p} & \cdots & a_{1N}\\ a_{21} & a_{22} & \cdots & \left(1-z_{2}\right)*a_{2p} & \cdots & a_{2N}\\ \vdots & \vdots & \ddots & \vdots & \ddots & \vdots \\ a_{M1} & a_{M2} & \cdots & \left(1-z_{M}\right)*a_{Mp} & \cdots & a_{MN} \end{array}\right]\left[\begin{array}{c} s_{1}\\ s_{2}\\ \vdots \\ s_{p}\\ \vdots \\ s_{N} \end{array}\right] \end{array}$$

Let $w_{i}=1-z_{i}=w+\Delta_{i},i=1,2,3\cdots ,M$, then Equation (19) can be rewritten as follows:

$$\left[\begin{array}{c} y_{e1}\\ y_{e2}\\ y_{e3}\\ \vdots \\ y_{eM} \end{array}\right]=\left[\begin{array}{ccccc} a_{11} & a_{12} & a_{13} & \cdots & a_{1N}\\ a_{21} & a_{22} & a_{23} & \cdots & a_{2N}\\ \vdots & \vdots & \vdots & \ddots & \vdots \\ a_{M1} & a_{M2} & a_{M3} & \cdots & a_{MN} \end{array}\right]\left[\begin{array}{c} s_{1}\\ s_{2}\\ \vdots \\ ws_{p}\\ \vdots \\ s_{N} \end{array}\right]+$$

(20) $$\left[\begin{array}{cccccc} 0 & 0 & \cdots & \frac{\Delta_{1}}{w}*a_{1p} & \cdots & 0\\ 0 & 0 & \cdots & \frac{\Delta_{2}}{w}*a_{2p} & \cdots & 0\\ \vdots & \vdots & \vdots & \vdots & \ddots & \vdots \\ 0 & 0 & \cdots & \frac{\Delta_{M}}{w}*a_{Mp} & \cdots & 0 \end{array}\right]\left[\begin{array}{c} s_{1}\\ s_{2}\\ \vdots \\ ws_{p}\\ \vdots \\ s_{N} \end{array}\right]$$

To further simplify the discussion, we assume that all $\Delta_{i}=0,i=1,2,3\cdots ,M$, so we get,

(21) $$y_{e}=As^{{}^{\prime }}$$

where $s^{{}^{\prime }}=\left[\begin{array}{c} s_{1}\\ s_{2}\\ \vdots \\ ws_{p}\\ \vdots \\ s_{N} \end{array}\right]$, $s^{{}^{\prime }}\in R^{N\times 1}$. When restricted-authorized receivers use the OMP algorithm with the input $y_{e}$ and *A*, they obtain the signal $s^{\prime }$ as a result.

For the normal case, if at least one $\Delta_{i}\neq 0$, then Equation (21) could be rewritten as,

(22) $$y_{e}=\left(A+\Delta A\right)s^{{}^{\prime }}$$

where $\Delta A=\left[\begin{array}{cccccc} 0 & 0 & \cdots & \frac{\Delta_{1}}{w}*a_{1p} & \cdots & 0\\ 0 & 0 & \cdots & \frac{\Delta_{2}}{w}*a_{2p} & \cdots & 0\\ \vdots & \vdots & \vdots & \vdots & \ddots & \vdots \\ 0 & 0 & \cdots & \frac{\Delta_{M}}{w}*a_{Mp} & \cdots & 0 \end{array}\right]$, and, as explained in [45], no reduction in the reconstruction can be achieved when the noise added to the measurement matrix is not arbitrarily large. Clearly, the boundedness characteristic of chaotic systems is an additional prerequisite of the successful reconstruction of signal $s^{\prime }$.

**Figure 3:**
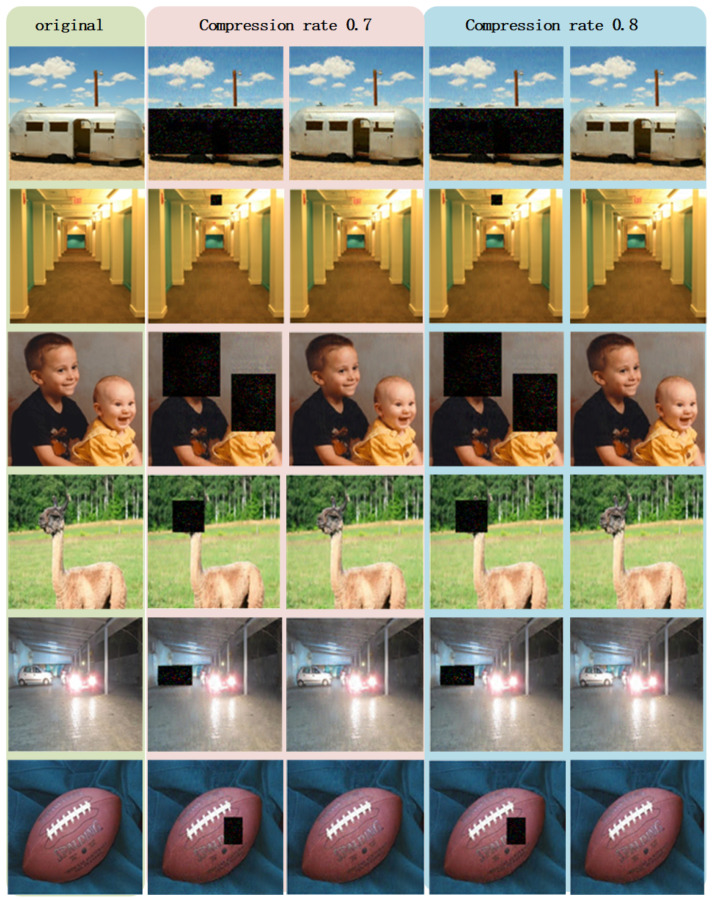
The first column shows the original images named trailer, hallway, kids, llama, car, and football, respectively. The second column shows the reconstructed images obtained by restricted-authorized receivers under compression rate 0.7. The third column shows the reconstructed images obtained by full-authorized receivers under compression rate 0.7. The fourth column shows the reconstructed images obtained by restricted-authorized receivers under compression rate 0.8. The fifth column shows the reconstructed images obtained by full-authorized receivers under compression rate 0.8.

We conduct a substantial number of experiments to verify the feasibility of the proposed method from a practical perspective. The results of several experiments using pictures from MATLAB R2020b image library are reported in Figure 3, along with the values of peak signal-to-noise ratios (PSNRs, dB) recorded in Table 1.

By observing the second and fourth columns of Figure 3, it is crystal clear that the assumed critical information in the original images is concealed, and other information contained in the original signals is reconstructed successfully. Simultaneously, from the third and fifth columns of Figure 3, we can obtain vivid reconstructions of the entire image without any concealment.

The PSNR values that are calculated based on pixels of the original images and the reconstructed images from red, green, and blue channels are listed in Table 1. From these figures, we surmise that, although the values of PSNR varies due to the difference between images and compression rates, all the PSNR values exceed 30 dB, which could be regarded as a benchmark for acceptable image reconstruction qualities.

**Figure 4:**
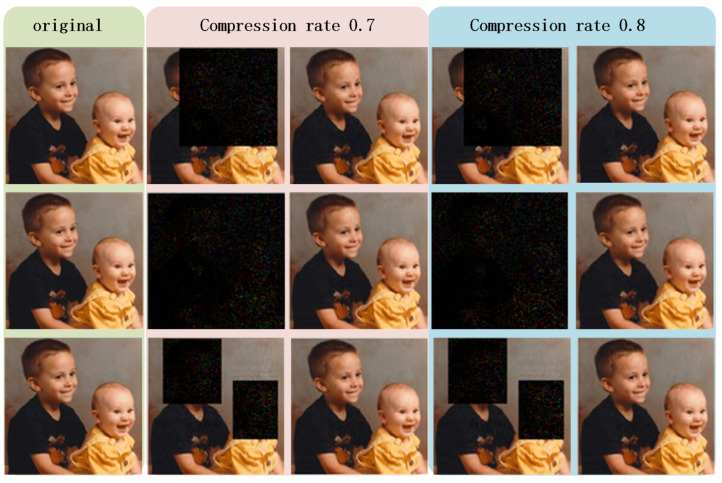
The concealed sections in the first and second rows are 50% and 100%, respectively, and the last row shows the results of concealing two independent sections of the original image. The first column contains the original image, the second and third columns show the reconstruction results of restricted-authorized receivers and full-authorized receivers under compression rate 0.7. The fourth and fifth columns show the reconstruction results under compression rate 0.8.

From Figure 4, we see that flexibility is achieved in terms of concealing portions and the number of concealed sections, and the related PSNR values in Table 2 all exceed 30 dB, which represents an acceptable quality of image reconstruction.

## 6. Robustness Analysis

To discuss the influence of noise during transmission and the robustness of the proposed method, the impact of random noise is analyzed. Here we assume that the noise in the transmission channel is white Gaussian noise, and from Equation (12) we get,

(23) $$y_{c}=\alpha y_{e}+\beta a_{j}+\delta_{G}$$

where $\delta_{G}\in R^{M\times 1}$ is a vector conformed to white Gaussian noise.

**Figure 5:**
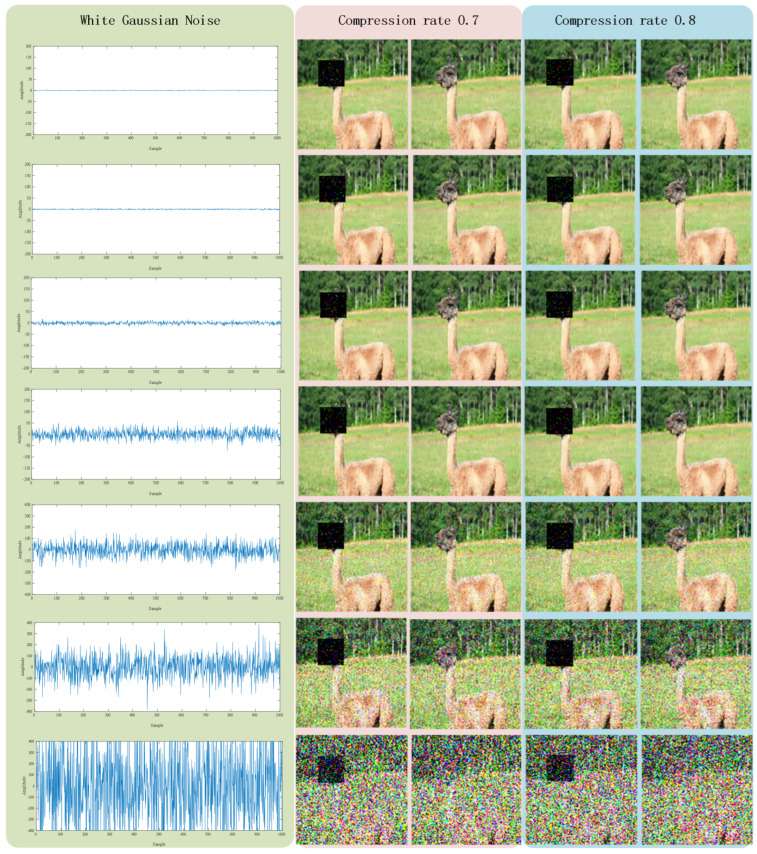
Reconstruction results with the impact of white Gaussian noise. The first column shows the noise with power values of 0, 5, 15, 25, 35, 40, and 50 in dBW, respectively. The second and third columns show the reconstruction results under compression rate 0.7, and the fourth and fifth columns show the reconstruction results under compression rate 0.8.

Figure 5 reports the reconstruction results under the impact of white Gaussian noise with different values of noise power. Table 3 lists the PSNR values under the circumstance with white Gaussian noise.

From Figure 5 and Table 3, we find that noise does have an impact on the quality of image reconstruction as well as the PSNR values. With increasing noise power, the impact gradually becomes stronger. When the power of noise is below 15 dBW, the quality of image reconstruction is almost unaffected, and the PSNR values decrease slightly, although they are still above 30. When the power of noise has reached 35 dBW, the quality of image reconstruction is still tolerable, although the values of PSNR are noticeably below 30. When the power of noise is higher than 40, the quality of image reconstruction seriously decreases, and the PSNR values also reduce significantly.

## 7. Security Analysis

After original signals are processed by the proposed method, even if attackers obtain the transmitted data, that is $Y_{c}$, by some sort of deviousness, it is highly likely that they cannot achieve the original signals under the current computing power level in a tolerable time duration, because in the proposed method signals are encrypted with a tremendous key space. Attackers could obtain very little useful information, because the signals operated by the proposed method leak very few statistics messages.

### 7.1. Chaotic Compressive Sensing Security Analysis

In a classical cryptosystem, assume the plaintext is *p*, the ciphertext is *c*, if P(c)=P(c|p), then the cryptosystem is considered to be secure [46]. For modern cryptosystems, they are often designed to be computationally secure. Namely, the cryptosystem could not be broken by existing sophisticated tools within polynomial time. Specifically, if an encryption scheme is sensitive to initial conditions and has tremendous secret key space, then we regard such an encryption scheme as a secure encryption scheme [47].

Chaotic systems are famous for sensitive dependence on initial conditions. When this characteristic is applied to encryption, it behaves as an entirely different decryption result, even if the secret key changes only slightly.

**Figure 6:**
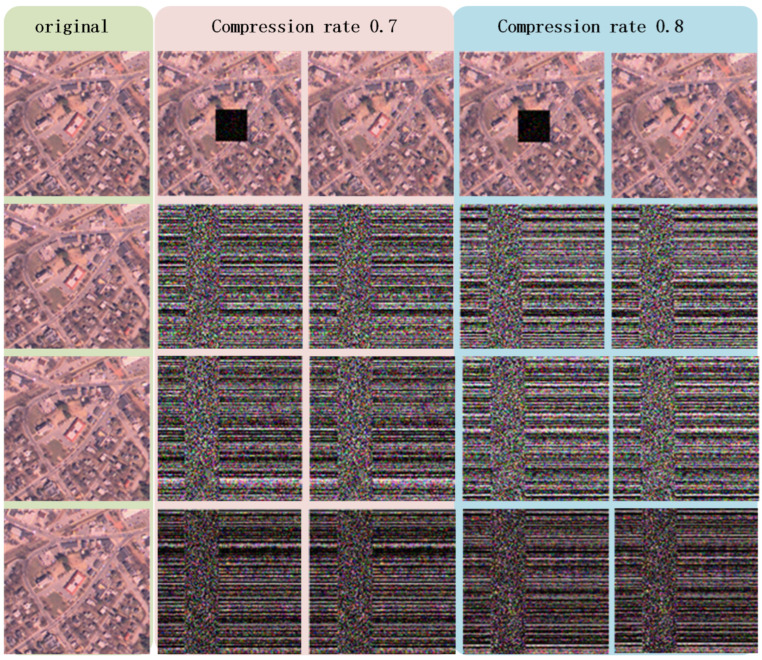
The first row shows the reconstruction results using the right initial value. The second row shows the reconstruction results using the initial value that is modified $10^{-15}$. The third row shows the reconstruction results using the initial value that is modified $10^{-16}$. The fourth row shows the reconstruction results using the initial value that is modified $10^{-17}$.

Figure 6 shows that, after a slight change is applied on the initial value of the chaotic sequence, that is $z_{0}^{{}^{\prime }}$, the experimental reconstruction results greatly change.

In the proposed method, there are four parameters participating in generating chaotic measurement matrices. The secret key A, which must be used by either restricted-authorized receivers or full-authorized receivers, is determined by chaotic parameter *b*, initial value $z_{0}^{{}^{\prime }}$, initial position $n_{0}$, and sampling distance *d*. Accordingly, we define $K_{b},K_{z},K_{n},K_{d}$, and the key space *K* is,

(24) $$K=K_{b}\times K_{z}\times K_{n}\times K_{d}.$$

Suppose we use a 32-bit processor in which the data precision of a double-precision floating point type is 16 significant digits after the decimal point, and suppose $K_{n}$ and $K_{d}$ are 100 and 10, respectively, then we can calculate from Equation (24) that $K\approx 10^{35}$ (See Table 4). Such a number could be enlarged by adjusting the value ranges of the parameters. For instance, if we change the value range of $K_{d}$ from [1, 10] to [1, 100], then the key space will be increased 10 times. In addition, increasing the precision of numbers that participate in operations could also be helpful to enlarge the key space of the proposed method. However, such an operation may increase the running time and complexity of the proposed method. Therefore, there should be a compromise when setting the range and number precision of parameters, according to the security and efficiency requirements.

### 7.2. Pixel Distribution Analysis

**Figure 7:**
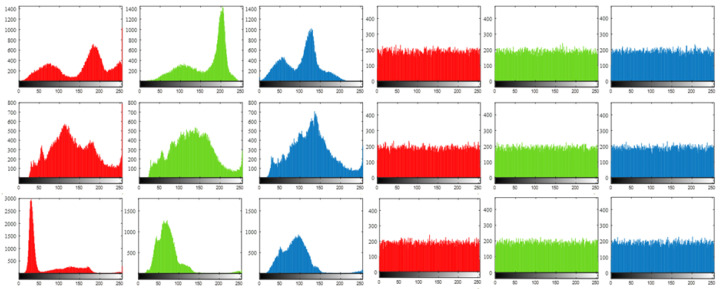
The first, second, and third columns show the histograms of pixel distribution that are calculated based on pixels from red, green, and blue channels of original RGB images that are named llama, car, and football, respectively. Correspondingly, the fourth, fifth, and sixth columns show the histograms of pixel distribution that are calculated based on pixels from signal $Y_{c}$. The compression rate is 0.7.

Statistical analysis attacks mean that attackers try to obtain the secret key by analyzing the statistical rules or patterns divulged from encrypted signals and their relevant original signals. In the Internet or wireless network environment, attackers might easily listen to the network flows to capture ciphertexts, which contain signal $Y_{c}$ of the proposed method. These malicious or just curious attackers might sum up the statistical laws and patterns revealed by the resource they have obtained and manage to extract the transformation relationships between plaintexts and ciphertexts, so as to analyze the encryption scheme. Figure 7 reports histograms of pixel distribution that are calculated based on pixels from red, green, and blue channels of original RGB images and histograms that are calculated based on pixels from red, green, and blue channels of signal $Y_{c}$, which may be transmitted through open or insecure channels.

Figure 7 implies that the pixel distribution of the original images leaks obvious statistical laws and patterns, whereas the histograms generated based on signal $Y_{c}$ clearly show that the pixels in such signals are distributed uniformly. It is noticeable that the histograms of pixel distribution of the original images and of signal $Y_{c}$ have no internal connections to each other. As is well known, the more uniform the pixel distribution is, the less statistical information the signal reveals, and the more secure the encryption scheme is.

### 7.3. Correlation Analysis

Correlation analysis refers to the analysis of values of two or more variables’ correlation, with the aim of measuring the correlation level between different variables. The correlation value is calculated by

(25) $$r_{XY}=\frac{cov(X,Y)}{\sqrt{D(X)}\sqrt{\left(D(Y\right)}}$$

where *X* and *Y* represent two variables. The mathematical expectations of *X* and *Y* are $E\left(X\right)=\frac{1}{N}\sum_{i=1}^{N}X_{i}$ and $E\left(Y\right)=\frac{1}{N}\sum_{i=1}^{N}Y_{i}$. The covariance between *X* and *Y* is $cov\left(X,Y\right)=\frac{1}{N}\sum_{i=1}^{N}\left(X_{i}-E\left(X\right)\right)\left(Y_{i}-E\left(Y\right)\right)$. The variances of *X* and *Y* are $D\left(X\right)=E\left(X^{2}\right)-{\left(E(X)\right)}^{2}$ and $D\left(Y\right)=E\left(Y^{2}\right)-{\left(E(Y)\right)}^{2}$.

Table 5, Table 6 and Table 7 list the correlation values of adjacent pixels of original images as well as of signal $Y_{c}$. Values are calculated based on pixel pairs from horizontal, vertical, and diagonal directions, respectively.

Table 5, Table 6 and Table 7 indicate that the correlation values of adjacent pixels of original images are approximate to 1, which means that a pixel of an original image is highly likely to leak information about the pixels surround it. Taking advantage of this feature, attackers may infer or predict a pixel value according to a known pixel value next to it and then apply even more to the recovery of the whole image. In addition, it also can be seen that all the correlation values of adjacent pixels of signal $Y_{c}$ are nearly 0, which means that the strong correlations of pixels in the original images are broken, and, therefore, attackers could use little information about adjacent pixels to launch statistical attacks.

**Figure 8:**
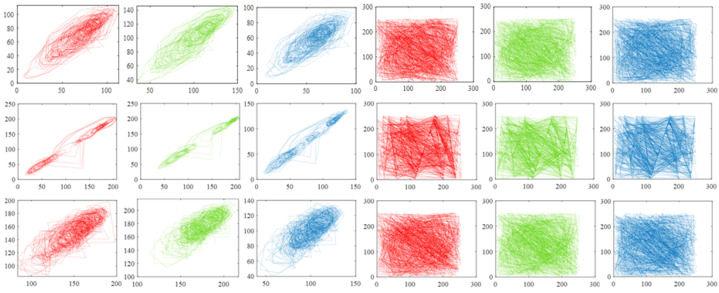
Correlation values of adjacent pixels of image llama. The first, second, and third columns are drawn based on adjacent pixels from the red, green, and blue channels of the original image, and the fourth, fifth, and sixth columns are drawn based on adjacent pixels of signal $Y_{c}$. The three rows show the results calculated from horizontal, vertical, and diagonal directions, respectively. The compression rate is 0.7.

**Figure 9:**
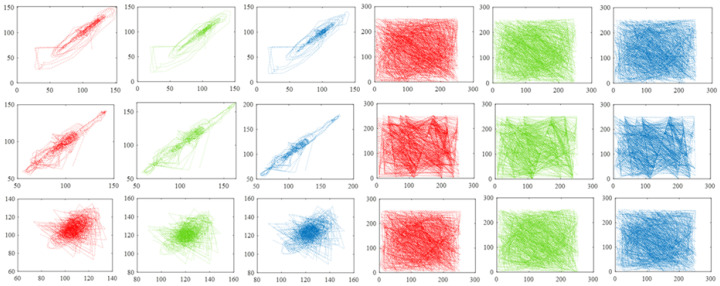
Correlation values of adjacent pixels of image car. The first, second, and third columns are drawn based on adjacent pixels from the red, green, and blue channels of the original image, and the fourth, fifth, and sixth columns are drawn based on adjacent pixels of signal $Y_{c}$. The three rows show the results calculated from horizontal, vertical, and diagonal directions, respectively. The compression rate is 0.7.

**Figure 10:**
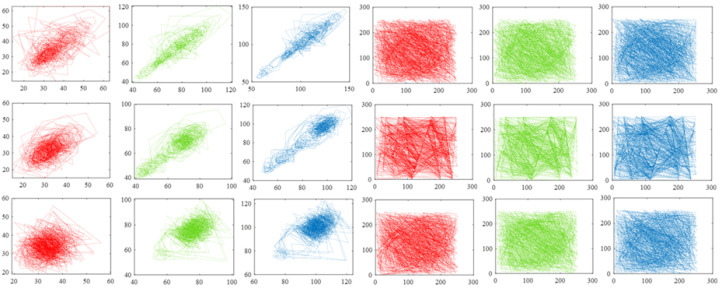
Correlation values of adjacent pixels of image football. The first, second, and third columns are drawn based on adjacent pixels from the red, green, and blue channels of the original image, and the fourth, fifth, and sixth columns are drawn based on adjacent pixels of signal $Y_{c}$. The three rows show the results calculated from horizontal, vertical, and diagonal directions, respectively. The compression rate is 0.7.

In addition, we randomly select 1000 pairs of adjacent pixels from the original image and related signal $Y_{c}$, respectively, and record pixel values through the coordinate system to reveal the correlation of adjacent pixels in another form. Figure 8 shows the results drawn based on image llama, which implies that the correlation of adjacent pixels in the original image is rather tough and the correlation of adjacent pixels in signal $Y_{c}$ is very weak. Figure 9 and Figure 10 exhibit the results generated by pixel pairs of image car, image football, and their related signal $Y_{c}$. Similarly, points drawn according to original pictures are distributed unevenly, which indicates a high value of pixel correlation, whereas points drawn from signal $Y_{c}$ are distributed arbitrarily, which means the value of pixel correlation of signal $Y_{c}$ is rather low.

Furthermore, we calculate the correlation of the pixels with exactly the same position chosen from the critical section of the original image and from its concealed form, and the results are reported in Table 8. All the values in Table 8 are extremely close to 0, which implies that the correlations of pixels between the original and concealed sections are low. In other words, the critical information in the original image is well protected.

### 7.4. Image Entropy Analysis

Commonly, entropy is defined to judge whether the complexity or randomness is strong enough. Information entropy plays an essential role in measuring randomness of information. Image entropy can act as a reference index of information randomness of an image. Each pixel in either channel r, channel g, or channel b of an RGB image has an intensity value or gray value between 0 to 255, and the ideal entropy value of such encrypted message is 8, which means that the information that is contained in such a massage is arbitrary. High values of image entropy also represent that the ability to resist statistical analysis. We use Equation (26) to calculate image entropy.

(26) $$H\left(x\right)=-\sum_{i=1}^{L}P(x_{i})\log_{2}P\left(x_{i}\right)$$

where $x_{i}\in 1,2,3,\cdots ,L$ represents the gray value of pixels, and $P\left(x_{i}\right),0\leq P(x_{i})\leq 1$, $\sum_{i=1}^{L}P(x_{i})=1$ is the probability of gray value $x_{i}$.

Table 9 lists the experimental results of information entropy of both original images and signal $Y_{c}$, and it implies that all the values calculated based on signal $Y_{c}$ are approaching 8, albeit with various entropy values of original images.

## 8. Discussion

In this section, we compare the compression performance of the proposed method with some recently proposed CS-based image processing methods [48,49,50]. It is worth noting that here we select methods that use chaotic measurement matrices or other types of measurement matrices that are generated by deterministic means, similar to the proposed methods.

**Figure 11:**
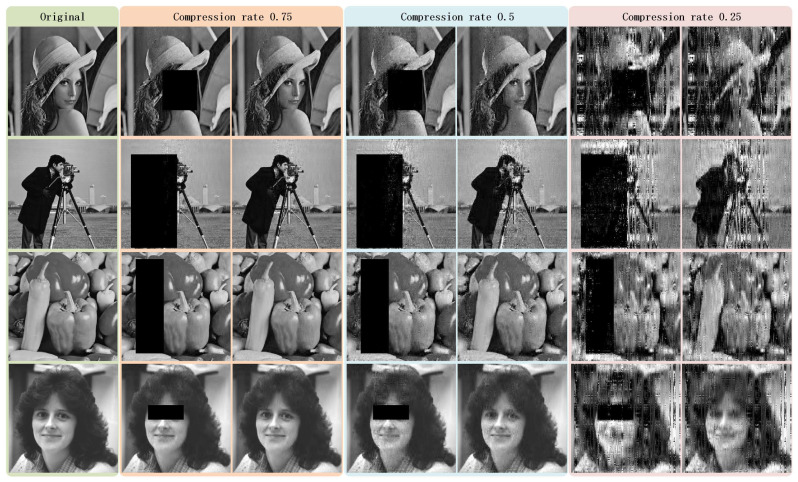
The first column shows the original images named lena, cameraman, peppers, and woman, respectively. The second, fourth, and sixth columns show the reconstructed images obtained by restricted-authorized receivers under compression rates 0.75, 0.5, and 0.25, respectively. The third, fifth, and seventh columns show the reconstructed images obtained by full-authorized receivers under compression rates 0.75, 0.5, and 0.25, respectively.

We conduct experiments using the same images used by [48,49,50]. The original images and their reconstruction results are exhibited in Figure 11. The related PSNR values are listed in Table 10. From data shown in Table 10, we can infer that, although the PSNR values vary when using different original images, the proposed method could archive similar reconstruction quality as Refs. [48,49,50], when the compression ratios reach 0.5. More importantly, the proposed method could achieve multi-level reconstruction for users in different groups. Namely, restricted-authorized users could merely reconstruct images with concealed critical information, whereas full-authorized users could reconstruct the entire images.

## 9. Conclusions

In this paper, we propose a secure and efficient BBN data transmission method that could accomplish critical information concealment and retrieval. Generally, BBN sensors are resource constrained, and CS-based methods are naturally suitable for these sensors, as CS can accomplish data compression while sampling, and this process just needs simple operations of addition and multiplication, which could achieve the aim of reducing energy consumption of sensors during data processing and transmitting. The experimental results show that the proposed methods could compress and encrypt the original data and render different reconstruction results to users in different authorization groups. Namely, users in restricted-authorized groups could only obtain reconstruction results with critical sectors concealed, whereas users in full-authorized groups could reconstruct entire data.

Moreover, in the proposed method, chaotic systems are introduced to generate measurement matrices, so the senders and receivers do not need to transmit the entire measurement matrices to one another, which further saves transmission energy. Specifically, the proposed method could enhance the security level of data transmission by breaking the statistical patterns of original data, providing large key space and sensitivity of the initial values, etc. The key space of the proposed method is discussed, and simulation results show that when even a slight change is applied to the initial value of the chaotic sequences, 10–15 to 10–17, for example, the experimental reconstruction results greatly change.

Last but not least, experimental results also show that the proposed method enables the senders to conceal critical information with flexibility in terms of proportions and quantities of the concealed sectors. In summary, the proposed method realizes the protection of critical information that may be transmitted within BBNs. In the future, the combination of information concealment and semi-tensor compressive sensing could be studied, in order to enhance efficiency and flexibility levels of data transmission catering to the coming requirements of appliances in BBNs and even in IoT.

## Figures and Tables

**Table 1:** PSNR values under compression rates 0.7 and 0.8 (Unit: dB).

Compression Rate 0.7				
**Image**	**Channel r**	**Channel g**	**Channel b**	**Average**
trailer	30.53562	31.50471	32.48743	31.50925
hallway	35.46249	35.70703	37.18625	36.11859
kids	33.41342	33.73252	33.98569	33.71054
llama	32.27819	32.25344	32.39616	32.30926
car	31.82822	31.75158	31.79017	31.78999
football	31.67541	31.59554	31.27668	31.51588
**Compression Rate 0.8**				
**Image**	**Channel r**	**Channel g**	**Channel b**	**Average**
trailer	30.10448	30.32083	31.74121	30.72217
hallway	34.85190	35.30434	36.45469	35.53697
kids	32.73238	33.06132	33.37811	33.05727
llama	31.45512	31.51228	31.61599	31.52780
car	31.44679	31.26573	31.26015	31.32422
football	30.78486	30.87273	30.58413	30.74724

**Table 2:** PSNR values for various concealing sections (Unit: dB).

Compression Rate 0.7				
**Concealing Section**	**Channel r**	**Channel g**	**Channel b**	**Average**
50%	32.59798	33.13968	33.23807	32.99191
100%	36.11858	36.11858	36.11858	36.11858
two sectors	33.41342	33.73252	33.98569	33.71054
**Compression Rate 0.8**				
**Image**	**Channel r**	**Channel g**	**Channel b**	**Average**
50%	33.30480	33.67279	33.93345	33.63701
100%	33.33252	33.75152	33.86508	33.64971
two sectors	33.41341	33.73252	33.98569	33.71054

**Table 3:** PSNR values with the impact of white Gaussian noise (Unit: dB).

Compression Rate 0.7				
**Concealing Section**	**Channel r**	**Channel g**	**Channel b**	**Average**
none	31.455124	31.512282	31.615987	31.527797
5	31.319548	31.435341	31.660291	31.471726
15	31.22286	31.284546	31.357155	31.288187
25	29.367135	29.356614	29.023638	29.249129
35	20.061426	19.881269	18.988067	19.643587
40	14.417344	14.175233	13.895409	14.162662
50	8.000954	8.06832	8.150955	8.073409
**Compression Rate 0.8**				
**Image**	**Channel r**	**Channel g**	**Channel b**	**Average**
none	32.278187	32.253436	32.396164	32.309262
5	32.221204	32.178524	32.447941	32.282556
15	32.018806	32.040822	32.220331	32.093319
25	30.019168p	30.058696	29.816343	29.937519
35	20.110191	19.999357	18.652514	19.587354
40	13.602667	13.480053	13.183436	13.422052
50	7.63013	7.679259	7.780847	7.696745

**Table 4:** Secret key space analysis.

Parameter Name	Parameter Type	Value Range	Key Space
*b*	double-precision floating point	(0,1)	$K_{b}\approx 1\times 10^{16}$
$z_{0}^{{}^{\prime }}$	double-precision floating point	(0,1)	$K_{z}\approx 1\times 10^{16}$
$n_{0}$	positive integer	[100,199]	$K_{n}=100$
*d*	positive integer	[1,10]	$K_{d}=10$

**Table 5:** Correlation values of adjacent pixels in image llama.

Pixels from Original Image				
**Direction**	**Channel r**	**Channel g**	**Channel b**	**Average**
horizontal	0.976468	0.962231	0.948301	0.962333
vertical	0.983566	0.979146	0.974502	0.979071
diagonal	0.983607	0.979149	0.974644	0.979133
**Pixels from $Y_{c}$**				
**Direction**	**Channel r**	**Channel g**	**Channel b**	**Average**
horizontal	0.013742	0.004258	0.001669	0.006556
vertical	0.018640	0.025289	0.025203	0.023044
diagonal	0.024711	0.030140	0.030343	0.028398

**Table 6:** Correlation values of adjacent pixels in image car.

Pixels from Original Image				
horizontal	0.982515	0.979078	0.977904	0.979832
vertical	0.979157	0.974894	0.974456	0.976169
diagonal	0.979092	0.974866	0.974478	0.976145
**pixels from $Y_{c}$**				
**Direction**	**Channel r**	**Channel g**	**Channel b**	**Average**
horizontal	0.003977	0.002792	0.000689	0.002486
vertical	0.022264	0.026053	0.029963	0.026093
diagonal	0.027555	0.031170	0.034890	0.031205

**Table 7:** Correlation values of adjacent pixels in image football.

Pixels from Original Image				
**Direction**	**Channel r**	**Channel g**	**Channel b**	**Average**
horizontal	0.981740	0.944225	0.951633	0.9591993
vertical	0.979666	0.940694	0.948486	0.956282
diagonal	0.979744	0.940788	0.948652	0.956395
**Pixels from $Y_{c}$**				
**Direction**	**Channel r**	**Channel g**	**Channel b**	**Average**
horizontal	0.005781	0.004287	0.002708	0.004259
vertical	0.034930	0.026071	0.029472	0.030501
diagonal	0.039611	0.030745	0.034106	0.034821

**Table 8:** Correlation values for concealing analysis. The compression rate is 0.7.

Image	Channel r	Channel g	Channel b	Average
trailer	0.078644	0.099305	0.088672	0.088874
hallway	0.052972	0.058863	0.051743	0.054526
kids	0.094172	0.112676	0.120774	0.109207
llama	0.090918	0.081103	0.127626	0.099882
car	0.107737	0.049603	0.063837	0.073726
football	0.048435	0.068405	0.051792	0.056211

**Table 9:** Experimental results of image entropy. The compression rate is 0.7.

Entropy of Original Image				
**Image**	**Channel r**	**Channel g**	**Channel b**	**Average**
trailer	7.6132	7.3457	7.1752	7.3780
hallway	7.1481	7.2374	6.704	7.0298
kids	7.2444	7.0481	6.8418	7.0447
llama	7.6599	7.2238	7.3275	7.4037
car	7.6715	7.6285	7.6061	7.6353
football	6.5350	6.6437	6.9785	6.7190
**Entropy of Signal $Y_{c}$**				
**Image**	**Channel r**	**Channel g**	**Channel b**	**Average**
trailer	7.9890	7.9886	7.9891	7.9889
hallway	7.9881	7.9885	7.9889	7.9885
kids	7.9879	7.9882	7.9886	7.9882
llama	7.9877	7.9884	7.9886	7.9882
car	7.9883	7.9883	7.9880	7.9882
football	7.9887	7.9882	7.9883	7.9884

**Table 10:** Compression performance comparison via PSNR values (Unit: dB).

Compression Rate 0.75				
**Image**	**Proposed Method**	**Ref. [48]**	**Ref. [49]**	**Ref. [50]**
lena	31.23	34.19	29.56	-
cameraman	28.90	30.85	28.93	-
peppers	32.51	-	-	31.25
woman	36.16	-	-	33.92
**Image**	**Proposed Method**	**Ref. [48]**	**Ref. [49]**	**Ref. [50]**
lena	25.79	29.82	29.82	-
cameraman	22.91	26.71	29.43	-
peppers	26.03	-	-	24.85
woman	31.16	-	-	30.82
**Compression Rate 0.25**				
**Image**	**Proposed Method**	**Ref. [48]**	**Ref. [49]**	**Ref. [50]**
lena	13.95	25.93	26.06	-
cameraman	15.07	22.64	25.23	-
peppers	17.80	-	-	19.16
woman	17.47	-	-	25.05

## Data Availability

Not applicable.

## Abbreviations

The following abbreviations are used in this manuscript:

BBN	Body to Body Network
IoT	Internet of Things
WBAN	Wireless Body Area Network
EEGs	Electroencephalograms
ECGs	Electrocardiograms
CS	Compressive Censing
WRAMC	Walter Reed Army Medical Center
MAC	Medium Access Control
RIP	Restricted Isometry Property
OMP	Orthogonal Matching Pursuit
LAN	Local Area Network
WAN	Wide Area Network
PSNR	Peak Signal-to-Noise Ratio
